# Air Pollution: Asia’s Two-Stroke Engine Dilemma

**DOI:** 10.1289/ehp.112-a613a

**Published:** 2004-08

**Authors:** Carol Potera

Asian cities face a serious air pollution problem from two- and three-wheeled vehicles that run on two-stroke engines. Global experts shared their knowledge about these vehicles at an international conference held 30 March–1 April 2004 at the Centre for Science and Environment (CSE) in Delhi, India. Anumita Roychowdhury, associate director of the CSE, said the inexpensive two-wheelers form a staggering 75–80% of the traffic in most Asian cities. She called them “an Asian dilemma.”

Because two-stroke engines burn an oil–gasoline mixture, they emit more smoke, carbon monoxide, hydrocarbons, and particulate matter than the gas-only four-stroke engines found in newer motorcycles. Making matters worse, many Asian two-wheelers are converted into three-wheeled “baby taxis” by adding a sidecar. However, “the vehicle is not designed for the extra weight, and the engine burns even dirtier,” said Michael Walsh, an independent consultant who advises nations worldwide about motor vehicle pollution and control issues.

The World Health Organization ranks urban outdoor air pollution as the thirteenth greatest contributor to disease burden and death worldwide. Air pollution raises the risk of respiratory illnesses; about two-thirds of the residents of Delhi and Calcutta suffer from respiratory symptoms such as common cold and dry and wet cough, which Twisha Lahiri, head of neuroendocrinology at India’s Chittaranjan National Cancer Institute, largely blames on two-stroke engine emissions.

In work presented at the conference, Lahiri and colleagues examined 2,000 non-smoking adults from Calcutta and Delhi and 300 from the rural Sunderban region, where air pollution is extremely low. Spirometry measurements found impaired lung function in 46% of Delhi adults and 56% of Calcutta adults, but only 21% of those from the Sunderban islands. Lahiri has also observed early indicators of lung cancer, such as metaplastic epithelial cells, in people exposed to traffic pollution. These findings “warrant immediate measures to abate the alarmingly high vehicular pollution in Indian cities,” she warned.

Measurements of how much pollution two-wheelers emit are rare, but one study of traffic intersections in Bangkok, Thailand, found that two-wheelers contributed up to 47% of particulates. When the city instituted a stringent inspection program and emissions standards in 2000, two-wheelers made up 96% of the city’s traffic; by March 2004 they made up only 40%, reported Supat Wangwongwatana, deputy director general of Thailand’s Pollution Control Department.

Similarly, when two-stroke baby taxis were phased out of Dhaka, Bangladesh, in 2002, particulate concentrations dropped up to 40%, and carbon monoxide and hydrocarbons fell significantly, reported S.M.A. Bari, director of engineering at the Bangladesh Road Transport Authority. However, no country has established particulate standards for two-wheelers, said Roychowdhury, and there are no standardized methods for measuring particulate emissions from these vehicles.

Economic incentives were what drove the transition from two-stroke to four-stroke tricycles in the Philippines’ San Fernando City. In 2001, three-quarters of the city’s 1,600 registered tricycles ran on two-stroke engines. The city council mandated a total phase-out of these vehicles by 2004 and offers interest-free loans for down-payments on four-stroke models. According to San Fernando City mayor Mary Jane Ortega, 400 four-stroke tricycles had replaced older two-stroke models as of March 2004.

The information presented at the conference supports public policies promoted by the CSE. “Small incremental steps will not help us beat the rapidly growing pollution,” said Roychowdhury. Instead, the CSE recommends stringent emissions standards for two-wheelers, an effective vehicle inspection program, fiscal incentive programs to replace existing two-stroke engines with four-stroke ones, and the development of efficient public transportation systems.

## Figures and Tables

**Figure:**
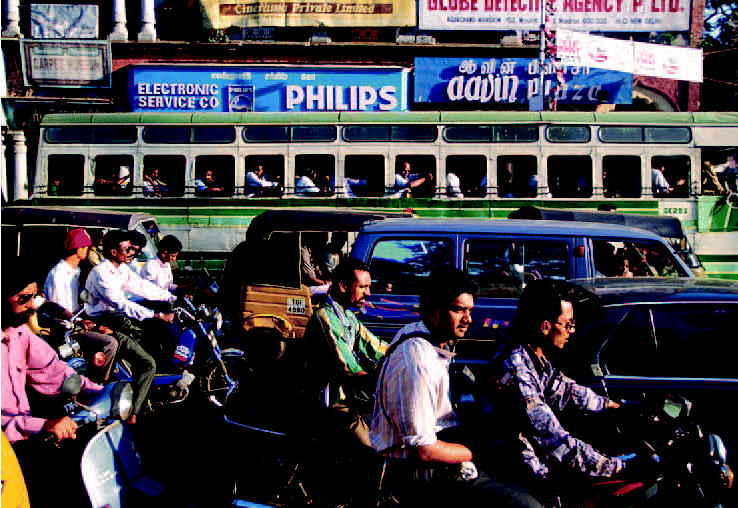
**Two strokes and you’re out.** Two-stroke engines, ubiquitous throughout Asia, are major contributors to air pollution and resulting respiratory illness in people.

